# *Primum non nocere. Quomo sapere?* [Firstly, do no harm. How can we know this?] Drug-eluting stents versus surgery

**DOI:** 10.1590/S1516-31802008000300001

**Published:** 2008-05-01

A recently published record of patients with multiarterial coronary disease in the State of New York who underwent angioplasty through emplacing drug-eluting stents, compared with patients treated with revascularization surgery, showed greater mortality in the group in which drug-eluting stents were used.^1^ In addition to the greater mortality, the incidence of myocardial infarct or the need for additional revascularization was also greater in the group treated with percutaneous intervention using drug-eluting stents.

At first sight, this is prime-quality evidence, since the study included 9,963 patients treated with drug-eluting stents and 7,437 with myocardial revascularization surgery. The follow-up lasted for around 18 months.^1^

These data reinforce the results from a systematic review with meta-analysis that was conducted by the Brazilian Cochrane Center for the Ministry of Health in 2004,^2^ as confirmed subsequently by other prospective studies and meta-analyses that demonstrated that bare-metal stents (cheaper) and drug-eluting stents were equivalent with regard to questions of mortality, incidence of infarct and need for surgical revascularization.^3–10^ Obviously, belief in the physiopathological theory that the consequences of local fibroblastic proliferation are reduced through the use of drug-eluting stents has waned in the light of the mounting evidence.

Another systematic review conducted by the team at the Brazilian Cochrane Center also showed that there was a lack of evidence that drug-eluting stents had better results in relation to the most important outcomes than did bare-metal stents stents, in diabetic patients.^3^

Some points should be made regarding the above information:

1. The effectiveness and **safety** of new technology need to be subjected to comparisons, before millions of lives and billions of dollars are involved. Inadequate conduct involving failure to do this has been repeated *ad infinitum* in Medicine.
2. Good medical science requires methodological rigor and absence of outside interests, emotions and fantasies, in order to reduce the uncertainties in decision-making and increase the effectiveness and efficiency. There need to be methodological and critical improvements among the professionals involved in such activities.
3. It is good strategy for the country and for our profession that the culture of practicing healthcare based on good evidence should permeate *all sectors* of healthcare decision-making and practice. The phrase “small mistakes with expensive new technologies” is almost a pleonasm, since almost all novelties reach requirements of around one billion reais per annum, from the Brazilian Ministry of Health, companies, patients and therefore taxpayers.
4. It is good to bear in mind that the Brazilian Ministry of Health invests around 30 billion reais per annum. Considering that hundreds of products containing new technology are launched on the market every year, generally based on extremely limited evidence that is of much poorer quality than what the use of drug-eluting stents was based on, there are insufficient financial resources to support this. And, the political and judicial pressures on the Ministry of Health and other administrative systems end up being very high.
5. This phenomenon does not occur only with manufactured products. A large proportion of physiotherapeutic and so-called alternative procedures are also not based on good scientific evidence. Clinical trials and systematic reviews are still required, for such procedures to be used more or be discarded on the basis of scientific proof.
6. Teaching of Evidence-based Medicine needs to be obligatory in all medical schools and in all health-related fields, because of the simple fact that it is necessary to separate efficiency and safety from waste and/or harmful action. This is increasingly a fundamental role among physicians.

In short, there are many other lessons to be learned from the enormous number of examples of mistakes that have historically been repeated in Medicine since its earliest days and which have ended up compromising the marvelous results from highly beneficial medical approaches. Moreover, some such methods have often been underutilized because of a lack of commercial interest in them.

However, this editorial cannot be concluded without pointing out that the comparative study on the treatment of heart diseased patients in the State of New York referred to at the start of this text was a retrospective *observational* study that only took into account the cases of patients who continued to live in the region. Many cases were lost from the follow-up, thereby weakening the evidence, since patients who disappear may have died, and this could change the results. Thus, the small differences in mortality and infarction rates might disappear.

Although the study sample was large and great effort was required to conduct the study, the level of evidence is lower than in a good clinical trial or a systematic review of clinical trials. Furthermore, the study in question did not incorporate comparisons with any equivalent group that only received clinical treatment. Therefore, it remains possible that surgery is better than or the same as drug-eluting stents, but we do not known by how much it would be superior to clinical treatment.

In other words, we have to adjust to practicing medicine based on the best evidence existing and continue to improve research methodology and our ability to critically assess new technologies. Through this, we will make fewer mistakes and can prepare new generations of teachers, researchers and healthcare professionals to face new technological challenges. The large numbers and seductive powers of such challenges tend to obscure decision-making by physicians, patients, managers, judges and ultimately all of society. In this respect, those who are responsible for capacitating new healthcare professionals have an extremely important role at the levels of undergraduate training and postgraduate research.

So what should be done? We remain with the conclusions from systematic reviews like the Cochrane reviews, which show that the use of drug-eluting stents reduces the incidence of restenosis in the treated vessel but does not reduce the rates of infarct or death, or the need for myocardial revascularization.

And the management of such cases? Whenever possible, decisions should be made by informed physicians and by patients who have been given explanations regarding the advantages and disadvantages of the options. If the patient is funding his own treatment, he may choose the most expensive option, if he believes that reducing the need for angioplasty has a worthwhile cost-benefit relationship.

Those who are responsible for public healthcare policies or health insurance policies may take the view that restenosis does not increase the risks of infarct, death or the need for revascularization surgery, and therefore there is no clinical or economic advantage that would justify the expenditure. They thus may invest the difference in better treatment for arterial hypertension, hypercholesterolemia, sedentarism, obesity and secondary prevention against infarct, using aspirin and other methods that are clearly efficient and safe. And the new technologies, as ever, will continue to be improved in order to attain the necessary effectiveness, efficiency and safety.

Speaking of this, a clinical trial published by Vlaar et al. in Lancet on June 7, 2008, reported that percutaneous aspiration of the coagulum in the coronary artery within the first hours of the infarction, when compared with expansion using a balloon (stents were placed in both groups), reduced mortality and the rate of recurrence of infarct by almost 50%, after one year of follow-up. There were 536 individuals in the aspiration group and other 536 in the control group. In other words, it seems that this study was a major progress at extremely low cost. Long live evidence-based technology!^11^
